# Barbiturate Is Not a Risk Factor For Late Ventilator Associated Pneumonia. a Post-Hoc Analysis On 441 Tbi Patients Included in 2 Rcts

**DOI:** 10.1186/2197-425X-3-S1-A483

**Published:** 2015-10-01

**Authors:** S Lasocki, K Asehnoune, P Seguin, A Roquilly, O Mimoz, Y Malledant, J-F Hamel, AtlanREA Group

**Affiliations:** LUNAM Université d'Angers, CHU d'Angers, Angers, France; CHU Nantes, Nantes, France; CHU Rennes, Rennes, France; CHU Poitiers, Poitiers, France

## Introduction

Barbiturate is a therapeutic option for the treatment of elevated intracranial pressure (eICP) in Traumatic brain injury (TBI) patients. It is used in 20% of the patients in a recent European cohort [[1]]. But, barbiturate may induce an immunosuppression. Indeed, barbiturate have been shown to induce a decrease in white blood cells in old reports [[2]] and to be associated to early ventilator associated pneumonia (VAP) [[3]]).

## Objectives

To assess if barbiturate is a risk factor for VAP in TBI patients.

## Methods

We conducted a post-hoc analysis of data obtained from 2 RCTs (Corti-TC and SPIRIT), in which use of barbiturate was prospectively collected. Occurrence of VAP, microbiologically confirmed, was the primary endpoint of these 2 RCTs. We conduct an univariate and multivariate analysis to identify risk factors associated with onset of early (≤7 days) or late (>7 days) VAP. Data are expressed as mean ± SD, medians (Q1-Q3) or Odd Ratio with (95% Confident Intervals).

## Results

Among the 441 severe TBI patients (age 39 ± 17 yrs, 135(80.3%) Male, SAPS II 44.7 ± 11.3, initial Glasgow score 5.7 ± 1.9, ISS 20.5 ± 13.7) included in the 2 RCTs (n = 275 for corti-TC and 166 for Spirit), 183(41%) experienced at least one episode of eICP (ICP>20 mmHg). Among these 183 patients, 138(75%) were treated with barbiturate (1[0-2] days after admission, for a duration of 3 (1-6) days in Corti-TC study). Characteristics of patients with eICP treated with barbiturate or not were not different, except for hyperventilation more often used in barbiturate patients (16(12%) vs 0 times, p = 0.017). in logistic regression, barbiturate use was the only factor associated with early VAP (OR 5.56 (95CI; 1.44-21.44), p = 0.013). the other factors included in the analysis were: gender, use of decompressive craniectomy, SAPS II score, initial Glasgow score, Head AIS Score, Tabaco use). This association with barbiturate use was not found for late onset VAP (OR 0.48 (0.17-1.39), p = 0.18). Only the initial head AIS score was associated with late VAP (OR 1.19 (1.21-3.02), p = 0.005). Patients treated with barbiturate had less ventilator free days at D28 (7 ± 8 days compared to 12 ± 9 for patients with eICP, p = 0.001, and to 13 ± 8 for the entire population, p = 0.001). Survival rate at day 28 was not different in barbiturate treated patients (71(60%) vs 14(64%) patients alive at D28, p = 0.72).

## Discussion

In these 2 recent RCTs, barbiturate use was an independent predictive factor for early VAP. This could be linked to the severity of the patients (despite the adjustments done. the absence of association with late pneumonia does not support a prolonged barbiturate induced immunosuppression. the ongoing longitudinal analysis, taking into account the time factor, will probably help to precise if barbiturate is a risk factor for early VAP or not.
